# ^18^F‐FDG PET in detection of primary age‐related tauopathy (PART) – Is there a role? Insights from an imaging‐pathology correlation study

**DOI:** 10.1002/alz.70568

**Published:** 2025-08-19

**Authors:** Anna Lavrova, Nha Trang Thu Pham, Cynthia J. Vernon, Jonathan Graff‐Radford, Bradley F. Boeve, David S. Knopman, Kejal Kantarci, Clifford R. Jack, Aivi T. Nguyen, Reichard R. Ross, Ronald C. Petersen, Dennis W. Dickson, Val Lowe, Jennifer L. Whitwell, Keith A Josephs

**Affiliations:** ^1^ Department of Radiology Mayo Clinic Rochester Minnesota USA; ^2^ Department of Neurology Mayo Clinic Rochester Minnesota USA; ^3^ Department of Laboratory Medicine and Pathology Mayo Clinic Rochester Minnesota USA; ^4^ Department of Neuroscience Mayo Clinic Mayo Clinic Jacksonville Florida USA

**Keywords:** ^18^F‐fluorodeoxyglucose positron emission tomography, argyrophilic grain disease, FDG‐PET, primary age‐related tauopathy, TAR DNA‐binding protein 43

## Abstract

**INTRODUCTION:**

Primary age‐related tauopathy (PART) is defined by neurofibrillary tangles (NFTs) with absent‐minimal amyloid beta (Aβ) plaques. Currently, definitive diagnosis of PART occurs with autopsy. This study investigated whether [^18^F]fluorodeoxyglucose positron emission tomography (FDG‐PET) could detect PART‐related metabolic changes and assessed the impact of common co‐pathologies.

**METHODS:**

We performed a retrospective cross‐sectional study of 88 individuals (mean age 85.6) with autopsy‐confirmed PART (Braak I to IV; Thal phases 0 to 2) who underwent *ante mortem* FDG‐PET. Visual ratings and standardized uptake value ratios (SUVRs) were analyzed in medial and lateral temporal lobes, inferior temporal pole, precuneus, and posterior cingulate regions.

**RESULTS:**

Medial temporal hypometabolism was observed in PART, in the presence of co‐pathologies. Argyrophilic grain disease and TAR DNA‐binding protein 43 were associated with greater hypometabolism (*p* < 0.01). Lewy body disease affected parietal regions.

**DISCUSSION:**

FDG‐PET reveals that PART‐related hypometabolism occurs when co‐pathologies are present, but PART alone appears to have minimal effect on medial temporal lobe hypometabolism.

**Highlights:**

- FDG‐PET hypometabolism worsens with Braak NFT stage.
- FDG‐PET detects mild lateral temporal lobe hypometabolism in PART alone.
- PART alone has a minimal effect on medial temporal lobe hypometabolism.
- Medial temporal hypometabolism is worse in PART with TDP‐43 and especially AGD.
- The presence of LBD contributes to parietal hypometabolism on FGD‐PET in PART.

## 1 BACKGROUND

Tau proteins, predominantly found in neurons, play a critical role in stabilizing microtubules and maintaining neuronal structure.^1^, ^2^ In pathological conditions such as Alzheimer's disease (AD) and other tauopathies, tau undergoes hyperphosphorylation, leading to toxic aggregates and neurofibrillary tangles (NFTs).^3^, ^4^ These alterations disrupt microtubules and affect signaling, leading to cognitive dysfunction and neurodegeneration.^5^, ^6^, ^7^ The Braak staging system was developed to categorize NFT distribution (Braak stages I and II start in the transentorhinal cortex, Braak stages III and IV involve limbic regions, and Braak stages V and VI include the neocortex).^8^

A condition similar to AD but characterized by the presence of NFTs without or with minimal amyloid beta (Aβ) deposition was introduced in 2014 as primary age‐related tauopathy (PART).^9^ Frequently observed in older adults,^10^, ^11^, ^12^ PART was previously referred to as “tangle‐only dementia” or “tangle‐predominant senile dementia” in the context of cognitive impairment with advanced tau pathology.^13^, ^14^ PART is subclassified as “definite” PART, characterized by an absence of Aβ senile plaques (Braak NFT stages I to IV, Thal phase 0), or “probable” PART, with sparse Aβ plaques (Thal phases 1 and 2).^9^ PART may be observed in isolation or coexist with other neurodegenerative diseases.^9^, ^15^, ^16^

Currently, the diagnosis of PART can only be confirmed through autopsy.^9^ Efforts to explore imaging techniques for *ante mortem* diagnosis have yielded limited specificity. Structural magnetic resonance imaging (MRI) in “definite” PART shows associations between Braak NFT staging and medial temporal lobe atrophy,^17^, ^18^ though anterior temporal atrophy may distinguish PART from AD.^19^ Another imaging modality, positron emission tomography (PET) with tau ligands, such as ^18^F‐flortaucipir (FTP), has been introduced as a potential diagnostic tool.^20^, ^21^ Flortaucipir demonstrates excellent binding to paired helical filament tau in NFTs in AD,^22^, ^23^, ^24^, ^25^, ^26^ aligning well with NFT severity and regional distribution per Braak NFT staging,^27^, ^28^, ^29^, ^30^ surpassing other tools in differentiating AD from other diseases.^31^ However, in PART, flortaucipir has limited sensitivity for detecting tau,^32^, ^33^ likely due to the absence of Aβ^34^ and/or differences in NFT composition (e.g., ghost tangles).^35^ In addition, off‐target binding of flortaucipir in the choroid plexus may interfere with signal quantification in adjacent medial temporal structures, such as the hippocampus, further complicating the accurate detection of tau pathology in PART.^22^

To our knowledge, no studies have explored whether PART is associated with specific hypometabolism patterns on ^18^F‐fluorodeoxyglucose (FDG) PET or its utility in detecting PART. Therefore, our main objective was to assess whether PART in isolation was associated with specific FDG‐PET alterations and characterize their nature and regional distribution. We also investigated the impact of co‐pathologies, including Lewy body disease (LBD), TDP‐43 proteinopathy, also known as limbic‐predominant age‐related TDP‐43 encephalopathy neuropathologic change (LATE‐NC) in the elderly, and argyrophilic grain disease (AGD), on FDG‐PET findings. While FDG‐PET is not routinely used in the clinical diagnosis of PART, this study was designed to assess its value as a research tool for understanding the metabolic correlates of neuropathology. The study emphasized the clinical utility of visually grading FDG‐PET findings, validated through quantitative standardized uptake value ratios (SUVRs) in the medial temporal lobe. Together, these findings give novel evidence of the metabolic alterations associated with PART and co‐pathologies, emphasizing that FDG‐PET warrants further investigation in clinical and research settings.

## 2 METHODS

### 2.1 Study design and participants

We identified individuals enrolled in the Mayo Clinic Neurodegenerative Research Group, Alzheimer's Disease Research Center, or Study of Aging who had undergone *ante mortem* FDG‐PET imaging and subsequently died with a completed brain autopsy (*n* = 248). From this cohort we selected cases with Braak NFT stages I to IV and no/sparse Aβ deposition (Thal phases 0 to 2) (*n* = 101). Individuals with Braak stage V or VI were not considered in order to avoid inclusion of cases with widespread neocortical tau pathology suggestive of AD or indicative of mutations in the microtubule‐associated protein, tau (MAPT), gene. Cases with frontotemporal lobar degeneration (FTLD), including FTLD‐tau and FTLD‐TDP, corticobasal degeneration, progressive supranuclear palsy, Pick disease, and globular glial tauopathy, were excluded (*n* = 13). This resulted in a final cohort of 88 individuals with PART, who died between December 12, 2010 and April 12, 2024. The cohort included 30 females, with a mean age at death of 85.6 years (± 9.5 years; range, 57.4 to 103.6 years).

Each participant underwent comprehensive neurological and neuropsychological assessments,^36^ including the Mini‐Mental State Examination (MMSE)^37^ and the Clinical Dementia Rating scale.^38^ Behavioral neurologists classified participants as being cognitively unimpaired, having mild cognitive impairment (MCI),^39^ or having dementia.^40^ Syndromic diagnoses further categorized those meeting dementia criteria.

### 2.2 Pathological and genetic analyses

All cases underwent a standardized pathological examination.^41^ Braak NFT staging^8^ was applied using anti‐tau antibodies (AT8, 1:1.000), while Thal phase^42^ was applied using Aβ antibodies (6F/3D, 1:10). Participants met PART criteria^9^ with low Braak NFT stages (I to IV)^8^, ^43^ and no/sparse Aβ deposition (Thal phases 0 to 2).^42^, ^44^, ^45^ Neuritic plaque burden was assessed using either modified Bielschowsky silver stain or thioflavin‐S staining methods and assigned a Consortium to Establish a Registry for Alzheimer's Disease (CERAD) score.^46^ LBD^47^ was diagnosed using α‐synuclein immunohistochemistry (LB509, 1:200). TDP‐43 pathology was screened in the amygdala and hippocampus using the pS409/410 antibody (1:5000).^48^, ^49^, ^50^, ^51^ Hippocampal sclerosis (HpScl) was diagnosed as neuronal loss in the subiculum and/or CA1 region of the hippocampus independent of TDP‐43 status.^52^, ^53^ Aging‐related tau astrogliopathy (ARTAG)^54^ was identified with AT8 or CP13 antibodies, starting in the medial temporal lobe and extending to other regions. AGD^55^ was evaluated using AT8 or CP13 staining to detect argyrophilic grains and related features such as oligodendrocytic coiled bodies.

Genomic DNA was extracted with the QIAamp DNA Mini Kit (Qiagen) for apolipoprotein E (*APOE*) ε genotyping^56^ using a modified amplification method, enzyme digestion, and gel electrophoresis.

### 2.3 Neuroimaging analysis

All participants underwent PET imaging using a GE PET/CT scanner (GE Healthcare, Milwaukee, WI, USA) and received 459 MBq (367 to 576 MBq) of FDG. FDG‐PET imaging was performed from 30 to 38 min after injection, with a total acquisition time of 8 min, using a dynamic protocol consisting of four 2‐min frames.^57^ Data were reconstructed into a 256×256 matrix with a pixel size of 1.0 mm. All participants also underwent 3T MRI^58^ with a magnetization‐prepared rapid gradient echo (MPRAGE) sequence (TR/TE/TI: 2300/3/900 ms; slice thickness: 1.2 mm).

**RESEARCH IN CONTEXT**

1. **Systematic review**: We reviewed PubMed and conference abstracts related to neuroimaging in PART. No validated imaging biomarker currently exists for the in vivo detection of PART, and diagnosis remains neuropathological. Prior work has not established whether FDG‐PET can detect metabolic changes associated with PART or distinguish it from related co‐pathologies.
2. **Interpretation**: We found subtle medial temporal hypometabolism on FDG‐PET in PART‐only cases and more prominent hypometabolism in PART with AGD and TDP‐43 proteinopathy, which likely enhance/drive the metabolic patterns observed in many PART cases, making the identification of PART‐specific imaging signatures more challenging.
3. **Future directions**: Future research should use larger, pathologically confirmed samples to disentangle the metabolic contributions of PART versus co‐pathologies. It is crucial to develop imaging biomarkers that distinguish PART from overlapping conditions due to different proteinopathies. Longitudinal imaging‐pathology studies are needed to validate FDG‐PET findings and explore early detection of PART‐specific metabolic decline.

**FIGURE 1:**
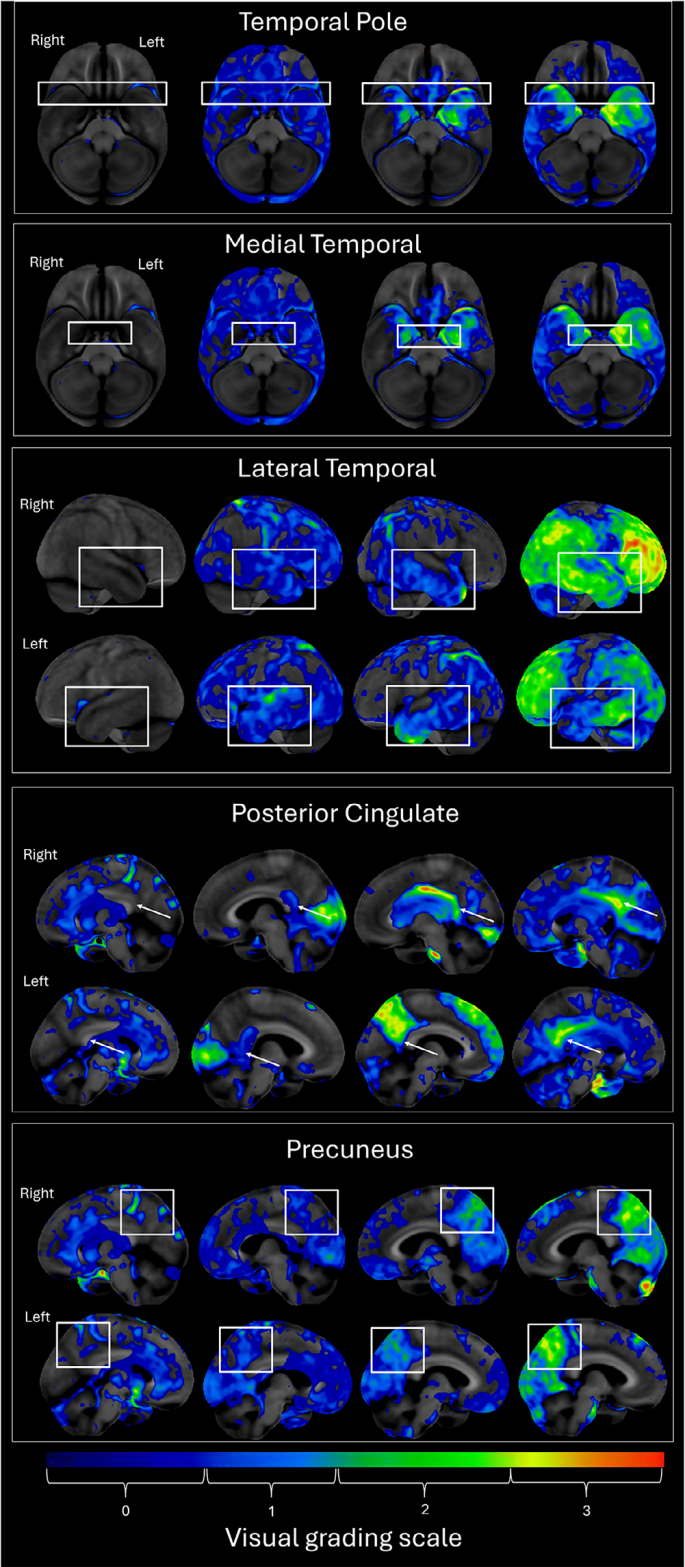
Visual grading scale for FDG‐PET hypometabolism. FDG‐PET images were processed using CortexID Suite and normalized to pons. Color overlays reflect *z*‐score deviations from age‐matched normative database. Regions were visually graded on a 0 to 3 scale based on hypometabolism severity. Colors represent *z‐*scores, not direct SUVR values. FDG‐PET, [^18^F]fluorodeoxyglucose positron emission tomography; SUVR, standard uptake value ratio.

FDG‐PET images were processed using the CortexID Suite (GE Healthcare, Waukesha, WI, USA). Images were normalized to pontine activity and compared against an age‐stratified normative database (*N* = 294, ages 30 to 89 years) to generate voxel‐wise *Z*‐score maps. Individual hypometabolism patterns were visualized using three‐dimensional stereotactic surface projections, and patterns in key regions (left and right temporal pole, medial temporal, lateral temporal, precuneus, and posterior cingulate regions) were visually graded by a radiologist (AL) on a 0 to 3 scale (Figure 1), where 0 = no hypometabolism, 1 = mild hypometabolism, 2 = moderate hypometabolism, and 3 = severe hypometabolism.^59^ Regions of interest were selected based on their known vulnerability to early NFT deposition in aging‐related tauopathies, including PART, as described in Braak stages I to IV.^9^ Visual scores were based on the presence and severity of focal hypometabolism; a higher score could be assigned if any portion of the region exceeded the *Z*‐score threshold, even if the remainder of the region showed relatively preserved uptake.

The Mayo Clinic Adult Lifespan Template was used to output medial temporal FDG uptake from the MPRAGE‐space FDG‐PET images. Median values in these regions were divided by median uptake in the pons to calculate SUVR.

### 2.4 Statistical analysis

Statistical analyses were performed using BlueSky Statistics version 10.3.1 and R version 4.4.1, with a significance threshold set at *p* ≤ 0.05. Chi‐squared and Kruskal–Wallis tests were used to compare categorical and continuous variables. Spearman rank correlations were conducted to evaluate the relationship between the number of affected regions versus the number of co‐pathologies and the severity of regional involvement versus the Braak NFT stage, as well as between visual scores and SUVRs. Regression models were employed to assess the influence of TDP‐43, LBD, HpScl, ARTAG, and AGD on medial temporal hypometabolism, using visual scores and SUVRs. Kruskal–Wallis test and analysis of variance (ANOVA) adjusted for the interval between FDG‐PET and death were performed to evaluate overall group differences in medial temporal SUVR values among the PART, PART+AGD, PART+TDP‐43, and PART+AGD+TDP‐43 groups, followed by post hoc comparisons using the Steel–Dwass test. False discovery rate (FDR) correction was applied to adjust for multiple comparisons where appropriate, with adjusted *p* values reported alongside unadjusted values.

## 3 RESULTS

### 3.1 Clinical and pathological characteristics by Braak NFT stage

Table 1 summarizes the clinical and pathological characteristics of the cohort stratified by Braak NFT stage (0 to IV). There were no significant group differences in age at death (*p* = 0.71), sex (*p* = 0.65), Thal Aβ phase (*p* = 0.47), APOE ε4 status (*p* = 0.12), or MMSE scores at the time of FDG‐PET (*p* = 0.66). Dementia diagnoses also did not differ significantly across Braak NFT stages (*p* = 0.29). TDP‐43, HpScl, ARTAG, and AGD pathology frequencies remained relatively stable across Braak NFT stages. LBD status, however, showed a trend toward higher frequency in those with higher NFT stage (*p* = 0.06), accompanied by a shift toward more advanced LBD stages. These results indicate that increasing Braak NFT stage alone was not associated with apparent clinical or demographic characteristic differences in this PART cohort.

**TABLE 1:** Clinical and pathological characteristics of PART study cohort stratified by Braak NFT stages.

Braak NFT stages						
	Braak 0 (*N* = 1)	Braak I (*N* = 13)	Braak II (*N* = 33)	Braak III (*N* = 30)	Braak IV (*N* = 9)	*p* value
Age at death						0.71 (1)
Median (Q1, Q3)	87.29 (87.29, 87.29)	81.68 (76.21, 88.11)	86.61 (79.98, 92.94)	88.48 (81.08, 92.46)	88.72 (83.59, 92.06)	
Sex						0.65 (2)
F	0 (0.0%)	6 (46.2%)	9 (27.3%)	12 (40.0%)	3 (33.3%)	
M	1 (100.0%)	7 (53.8%)	24 (72.7%)	18 (60.0%)	6 (66.7%)	
Thal phase						0.25 (2)
0	1 (100.0%)	10 (76.9%)	13 (39.4%)	12 (40.0%)	4 (44.4%)	
1	0 (0.0%)	1 (7.7%)	11 (33.3%)	11 (36.7%)	1 (11.1%)	
2	0 (0.0%)	2 (15.4%)	9 (27.3%)	7 (23.3%)	4 (44.4%)	
CERAD						0.72 (2)
Normal	1 (100.0%)	11 (84.6%)	20 (60.6%)	16 (53.3%)	5 (55.6%)	
Mild	0 (0.0%)	2 (15.4%)	10 (30.3%)	7 (23.3%)	2 (22.2%)	
Moderate	0 (0.0%)	0 (0.0%)	3 (9.1%)	6 (20.0%)	2 (22.2%)	
Frequent	0 (0.0%)	0 (0.0%)	0 (0.0%)	1 (3.3%)	0 (0.0%)	
Age at FDG‐PET						0.77 (1)
Median (Q1, Q3)	86.00 (86.00, 86.00)	79.57 (73.51, 84.54)	80.81 (76.38, 87.58)	81.56 (76.43, 87.50)	82.61 (80.44, 87.48)	
Time between FDG‐PET and death (y)						0.38 (1)
Median (Q1, Q3)	1.29 (1.29, 1.29)	2.48 (1.75, 6.24)	4.04 (2.55, 8.15)	4.04 (3.24, 7.34)	3.96 (3.15, 7.04)	
MMSE at FDG‐PET						0.66 (1)
Median (Q1, Q3)	29.00 (29.00, 29.00)	27.00 (23.00, 29.00)	28.00 (25.00, 29.00)	28.00 (25.25, 29.00)	27.50 (26.75, 29.00)	
Dementia (clinical)						0.29 (2)
no	1 (100.0%)	5 (38.5%)	22 (66.7%)	18 (60.0%)	7 (77.8%)	
yes	0 (0.0%)	8 (61.5%)	11 (33.3%)	12 (40.0%)	2 (22.2%)	
Clinical diagnosis						0.55 (2)
Atypical Alzheimer's dementia	0 (0.0%)	1 (7.7%)	0 (0.0%)	0 (0.0%)	1 (11.1%)	
Typical Alzheimer's dementia	0 (0.0%)	0 (0.0%)	3 (9.1%)	1 (3.3%)	0 (0.0%)	
Dementia – Unclassifiable/Mokri syndrome	0 (0.0%)	1 (7.7%)	1 (3.0%)	0 (0.0%)	0 (0.0%)	
Dementia with Lewy bodies	0 (0.0%)	2 (15.4%)	4 (12.1%)	5 (16.7%)	1 (11.1%)	
Mild cognitive impairment	0 (0.0%)	4 (30.8%)	3 (9.1%)	6 (20.0%)	0 (0.0%)	
Cognitively normal	1 (100.0%)	5 (38.5%)	22 (66.7%)	18 (60.0%)	7 (77.8%)	
*APOE*						0.12 (2)
Negative	0 (0.0%)	12 (92.3%)	26 (81.2%)	27 (90.0%)	7 (77.8%)	
Positive	1 (100.0%)	1 (7.7%)	6 (18.8%)	3 (10.0%)	2 (22.2%)	
TDP‐43 status						0.30 (2)
Negative	0 (0.0%)	11 (84.6%)	28 (84.8%)	24 (80.0%)	7 (77.8%)	
Positive	1 (100.0%)	2 (15.4%)	5 (15.2%)	6 (20.0%)	2 (22.2%)	
HpScl status						0.82 (2)
Negative	1 (100.0%)	12 (92.3%)	27 (81.8%)	27 (90.0%)	8 (88.9%)	
Positive	0 (0.0%)	1 (7.7%)	6 (18.2%)	3 (10.0%)	1 (11.1%)	
ARTAG status						0.41 (2)
Negative	0 (0.0%)	11 (84.6%)	21 (65.6%)	21 (70.0%)	6 (66.7%)	
Positive	1 (100.0%)	2 (15.4%)	11 (34.4%)	9 (30.0%)	3 (33.3%)	
AGD status						0.83 (2)
Negative	1 (100.0%)	11 (84.6%)	27 (81.8%)	24 (80.0%)	6 (66.7%)	
Positive	0 (0.0%)	2 (15.4%)	6 (18.2%)	6 (20.0%)	3 (33.3%)	
LBD status						0.06 (2)
Negative	0 (0.0%)	8 (61.5%)	26 (78.8%)	17 (56.7%)	3 (33.3%)	
Positive	1 (100.0%)	5 (38.5%)	7 (21.2%)	13 (43.3%)	6 (66.7%)	
LBD stage						0.08 (2)
Amygdala predominant	0 (0.0%)	1 (7.7%)	3 (9.1%)	5 (16.7%)	1 (11.1%)	
Brainstem predominant	1 (100.0%)	2 (15.4%)	1 (3.0%)	3 (10.0%)	3 (33.3%)	
Diffuse/neocortical	0 (0.0%)	1 (7.7%)	3 (9.1%)	5 (16.7%)	2 (22.2%)	
Limbic/transitional	0 (0.0%)	1 (7.7%)	0 (0.0%)	0 (0.0%)	0 (0.0%)	
None	0 (0.0%)	8 (61.5%)	26 (78.8%)	17 (56.7%)	3 (33.3%)	

*Note*: (1) Kruskal–Wallis rank sum test, (2) Pearson chi‐squared test. Data are shown as N (%) or median (Q1, Q3).

Abbreviations: AGD, argyrophilic grain disease; APOE, apolipoprotein E; ARTAG, age‐related tau astrogliopathy; CERAD, Consortium to Establish a Registry for Alzheimer's Disease; FDG‐PET, positron emission tomography with 18F‐fluorodeoxyglucose; HpScl, hippocampal sclerosis; LBD, Lewy body disease; MCI, mild cognitive impairment; MMSE, Mini‐Mental State Examination; PART, primary age‐related tauopathy; TDP‐43, trans‐active response DNA‐binding protein of 43 kDa.

### 3.2 Regional visual gradings by co‐pathologies

Figure S1 provides examples of typical patterns of FDG‐PET hypometabolism by co‐pathology. Table 2 summarizes the associations between regional severity scores and the presence of co‐pathologies. The left medial temporal lobe showed significant uncorrected associations with both TDP‐43 (*p *= 0.02, FDR‐adjusted *p *= 0.15) and AGD (*p *< 0.01, FDR‐adjusted *p *= 0.05), though only the association with AGD remained significant after FDR correction. Similarly, the right medial temporal lobe was associated with TDP‐43 (*p *= 0.03, FDR‐adjusted *p *= 0.15) and AGD (*p *< 0.01, FDR‐adjusted *p *= 0.05), with only AGD surviving correction.

**TABLE 2:** Chi‐squared test (for categorical variables) between severity of each assessed region vs. TDP‐43, AGD, LBD, HpScl status.

Region	TDP‐43 status				AGD status				LBD status				HpSc status			
	0 (*N* = 70)	1 (*N* = 16)	*p* value	FDR‐adjusted *p* value	0 (*N* = 69)	1 (*N* = 17)	*p* value	FDR‐adjusted *p‐*value	0 (*N* = 54)	1 (*N* = 32)	*p* value	FDR‐adjusted *p* value	0 (*N* = 75)	1 (*N* = 11)	*p* value	FDR‐adjusted *p* value
Temporal pole left			0.38	0.48			0.71	0.71			0.49	0.61			0.68	0.97
0	46 (65.7%)	8 (50.0%)			44 (63.8%)	10 (58.8%)			37 (68.5%)	17 (53.1%)			48 (64.0%)	6 (54.5%)		
1	17 (24.3%)	5 (31.2%)			18 (26.1%)	4 (23.5%)			11 (20.4%)	11 (34.4%)			19 (25.3%)	3 (27.3%)		
2	5 (7.1%)	3 (18.8%)			6 (8.7%)	2 (11.8%)			5 (9.3%)	3 (9.4%)			6 (8.0%)	2 (18.2%)		
3	2 (2.9%)	0 (0.0%)			1 (1.4%)	1 (5.9%)			1 (1.9%)	1 (3.1%)			2 (2.7%)	0 (0.0%)		
Temporal pole right			0.27	0.39			0.29	0.41			0.1	0.14			0.34	0.68
0	39 (55.7%)	7 (43.8%)			40 (58.0%)	6 (35.3%)			33 (61.1%)	13 (40.6%)			40 (53.3%)	6 (54.5%)		
1	24 (34.3%)	8 (50.0%)			24 (34.8%)	8 (47.1%)			15 (27.8%)	17 (53.1%)			28 (37.3%)	4 (36.4%)		
2	6 (8.6%)	0 (0.0%)			4 (5.8%)	2 (11.8%)			5 (9.3%)	1 (3.1%)			6 (8.0%)	0 (0.0%)		
3	1 (1.4%)	1 (6.2%)			1 (1.4%)	1 (5.9%)			1 (1.9%)	1 (3.1%)			1 (1.3%)	1 (9.1%)		
Precuneus left			0.23	0.38			0.08	0.20			**< 0.01**	**0.02**			0.87	0.99
0	39 (55.7%)	5 (31.2%)			38 (55.1%)	6 (35.3%)			36 (66.7%)	8 (25.0%)			39 (52.0%)	5 (45.5%)		
1	18 (25.7%)	6 (37.5%)			15 (21.7%)	9 (52.9%)			15 (27.8%)	9 (28.1%)			20 (26.7%)	4 (36.4%)		
2	11 (15.7%)	5 (31.2%)			14 (20.3%)	2 (11.8%)			3 (5.6%)	13 (40.6%)			14 (18.7%)	2 (18.2%)		
3	2 (2.9%)	0 (0.0%)			2 (2.9%)	0 (0.0%)			0 (0.0%)	2 (6.2%)			2 (2.7%)	0 (0.0%)		
Precuneus right			0.11	0.28			0.05	0.17			**< 0.01**	**0.02**			0.64	0.97
0	39 (55.7%)	5 (31.2%)			38 (55.1%)	6 (35.3%)			36 (66.7%)	8 (25.0%)			40 (53.3%)	4 (36.4%)		
1	18 (25.7%)	9 (56.2%)			17 (24.6%)	10 (58.8%)			16 (29.6%)	11 (34.4%)			22 (29.3%)	5 (45.5%)		
2	11 (15.7%)	2 (12.5%)			12 (17.4%)	1 (5.9%)			2 (3.7%)	11 (34.4%)			11 (14.7%)	2 (18.2%)		
3	2 (2.9%)	0 (0.0%)			2 (2.9%)	0 (0.0%)			0 (0.0%)	2 (6.2%)			2 (2.7%)	0 (0.0%)		
Medial temporal left			0.02	0.15			**< 0.01**	**0.05**			0.9	0.9			< 0.01	0.1
0	43 (61.4%)	7 (43.8%)			45 (65.2%)	5 (29.4%)			32 (59.3%)	18 (56.2%)			47 (62.7%)	3 (27.3%)		
1	20 (28.6%)	4 (25.0%)			18 (26.1%)	6 (35.3%)			14 (25.9%)	10 (31.2%)			20 (26.7%)	4 (36.4%)		
2	7 (10.0%)	3 (18.8%)			6 (8.7%)	4 (23.5%)			7 (13.0%)	3 (9.4%)			8 (10.7%)	2 (18.2%)		
3	0 (0.0%)	2 (12.5%)			0 (0.0%)	2 (11.8%)			1 (1.9%)	1 (3.1%)			0 (0.0%)	2 (18.2%)		
Medial temporal right			0.03	0.15			**0.01**	**0.05**			0.62	0.69			0.03	0.13
0	52 (74.3%)	7 (43.8%)			52 (75.4%)	7 (41.2%)			39 (72.2%)	20 (62.5%)			54 (72.0%)	5 (45.5%)		
1	15 (21.4%)	7 (43.8%)			15 (21.7%)	7 (41.2%)			12 (22.2%)	10 (31.2%)			18 (24.0%)	4 (36.4%)		
2	3 (4.3%)	1 (6.2%)			2 (2.9%)	2 (11.8%)			2 (3.7%)	2 (6.2%)			3 (4.0%)	1 (9.1%)		
3	0 (0.0%)	1 (6.2%)			0 (0.0%)	1 (5.9%)			1 (1.9%)	0 (0.0%)			0 (0.0%)	1 (9.1%)		
Lateral temporal left			0.91	0.91			0.49	0.54			**0.01**	**0.02**			0.9	0.99
0	34 (48.6%)	8 (50.0%)			36 (52.2%)	6 (35.3%)			32 (59.3%)	10 (31.2%)			36 (48.0%)	6 (54.5%)		
1	24 (34.3%)	6 (37.5%)			22 (31.9%)	8 (47.1%)			18 (33.3%)	12 (37.5%)			26 (34.7%)	4 (36.4%)		
2	10 (14.3%)	2 (12.5%)			9 (13.0%)	3 (17.6%)			4 (7.4%)	8 (25.0%)			11 (14.7%)	1 (9.1%)		
3	2 (2.9%)	0 (0.0%)			2 (2.9%)	0 (0.0%)			0 (0.0%)	2 (6.2%)			2 (2.7%)	0 (0.0%)		
Lateral temporal right			0.78	0.87			0.38	0.48			**< 0.01**	**0.02**			0.99	0.99
0	32 (45.7%)	7 (43.8%)			33 (47.8%)	6 (35.3%)			31 (57.4%)	8 (25.0%)			34 (45.3%)	5 (45.5%)		
1	30 (42.9%)	8 (50.0%)			28 (40.6%)	10 (58.8%)			21 (38.9%)	17 (53.1%)			33 (44.0%)	5 (45.5%)		
2	8 (11.4%)	1 (6.2%)			8 (11.6%)	1 (5.9%)			2 (3.7%)	7 (21.9%)			8 (10.7%)	1 (9.1%)		
3	N/A	N/A			N/A	N/A			N/A	N/A			N/A	N/A		
Posterior cingulate left			0.14	0.28			0.2	0.35			**< 0.01**	**0.02**			0.07	0.18
0	41 (58.6%)	7 (43.8%)			40 (58.0%)	8 (47.1%)			38 (70.4%)	10 (31.2%)			43 (57.3%)	5 (45.5%)		
1	23 (32.9%)	7 (43.8%)			24 (34.8%)	6 (35.3%)			14 (25.9%)	16 (50.0%)			26 (34.7%)	4 (36.4%)		
2	6 (8.6%)	1 (6.2%)			5 (7.2%)	2 (11.8%)			1 (1.9%)	6 (18.8%)			6 (8.0%)	1 (9.1%)		
3	0 (0.0%)	1 (6.2%)			0 (0.0%)	1 (5.9%)			1 (1.9%)	0 (0.0%)			0 (0.0%)	1 (9.1%)		
Posterior cingulate right			0.07	0.23			0.21	0.35			**< 0.01**	**0.02**			0.04	0.13
0	42 (60.0%)	7 (43.8%)			41 (59.4%)	8 (47.1%)			40 (74.1%)	9 (28.1%)			44 (58.7%)	5 (45.5%)		
1	23 (32.9%)	8 (50.0%)			24 (34.8%)	7 (41.2%)			12 (22.2%)	19 (59.4%)			26 (34.7%)	5 (45.5%)		
2	5 (7.1%)	0 (0.0%)			4 (5.8%)	1 (5.9%)			1 (1.9%)	4 (12.5%)			5 (6.7%)	0 (0.0%)		
3	0 (0.0%)	1 (6.2%)			0 (0.0%)	1 (5.9%)			1 (1.9%)	0 (0.0%)			0 (0.0%)	1 (9.1%)		

LBD demonstrated broader regional effects involving the bilateral precuneus, lateral temporal lobes, and bilateral posterior cingulate (*p* ≤ 0.01, FDR‐adjusted *p *= 0.02), all remaining significant after FDR correction. However, no association with medial temporal hypometabolism was observed for LBD (left: *p *= 0.90; right: *p *= 0.62).

HpScl showed uncorrected associations with the left (*p *< 0.01, FDR‐adjusted *p *= 0.10) and right (*p *= 0.03, FDR‐adjusted *p *= 0.13) medial temporal lobes and the right posterior cingulate (*p *= 0.04, FDR‐adjusted *p *= 0.13); none of these survived FDR correction.

In an ordinal regression analysis where all co‐pathologies are entered as predictors in the model, AGD status was associated with left (*p *= 0.001) and right (*p *= 0.004) medial temporal lobe scores, but TDP‐43 was not. HpScl status showed significance in the left medial temporal lobe (*p *= 0.024). LBD demonstrated associations with several regions, including bilateral lateral temporal lobes (left *p *= 0.002, right *p *= 0.001), bilateral posterior cingulate (left *p *= 0.001, right *p *< 0.001), and bilateral precuneus (left and right *p *< 0.001).

### 3.3 Clinical and pathological characteristics by number of affected regions

Table S1 summarizes the clinical and pathological characteristics of the study cohort, stratified by the number of visually identified FDG‐PET affected brain regions (score ≥ 1): no regions (*n* = 15), one to five regions (*n* = 37), and more than five regions (*n* = 34). Braak NFT staging revealed a significant association, with higher stages more frequently observed in individuals with a greater number of affected regions (*p *= 0.01). This was further supported by ordinal logistic regression adjusting for age at FDG‐PET (Table S2), where both the one‐ to five‐region group and over‐five‐region group showed significantly increased odds of having a higher Braak stage (OR = 6.23 and 7.83, respectively; *p* ≤ 0.002).

Cognitive status differed across groups, with the “more than five” category exhibiting lower median MMSE scores at the time of FDG‐PET (*p *< 0.01). In linear regression models adjusted for age (Table S3), having more than five affected regions was associated with significantly lower MMSE scores (β = −2.87; *p *= 0.015), suggesting that extensive hypometabolism is linked to greater cognitive impairment.

Dementia prevalence was highest among participants with more than five affected regions (52.9%), and the occurrence of dementia with Lewy bodies (DLB) was more common in this group (*p *= 0.03). Multinomial logistic regression (Table S4) confirmed these findings: Regional hypometabolism was strongly associated with a DLB clinical diagnosis (OR = 701.34; *p *= 0.002), while individuals with no dementia or with non‐DLB dementia types were less likely to have multiple affected regions (*p *< 0.01).

LBD status and stage were also closely related to the extent of FDG‐PET abnormalities. Logistic regression analysis (Table S5) showed that the odds of LBD diagnosis increased with the number of affected regions (OR = 13.70 for one to five regions, OR = 48.25 for over five regions; *p* ≤ 0.012), and ordinal regression (Table S6) revealed that greater LBD stage was associated with broader hypometabolism (*p *< 0.001). These associations were independent of age.

Cases with co‐pathologies were increasingly frequent as the number of affected regions rose (*p *< 0.01). A Spearman correlation analysis identified a moderate positive association between the number of co‐pathologies and the number of affected regions (ρ = 0.36, *p *= 0.0008) (Figure S2).

There were no significant differences across groups in age at death, sex, Thal Aβ phase, CERAD scores, or the presence of *APOE ε4*, TDP‐43, HpScl, ARTAG, or AGD (*p* > 0.05).

### 3.4 Clinical and pathological characteristics by number of co‐pathologies

Across participants stratified by number of co‐pathologies (zero to five) (Table S7), significant group differences were observed in MMSE performance (*p *= 0.04) and dementia subtype distribution (*p* < 0.01). Median MMSE declined from 28.0 (Q1:27.0, Q3:29.0) in the zero‐pathology group to 23.0 in the five‐pathology group, suggesting that increased co‐pathological burden was associated with greater cognitive impairment.

The dementia subtype also varied by co‐pathology count. DLB was most common in those with one or two co‐pathologies, while dementia of the Alzheimer's type (DAT) was present only in those with at least three co‐pathologies. Participants without dementia were primarily concentrated in the zero‐ to two‐pathology groups and were not present in the four‐ to five‐pathology groups.

Other variables – including age at death (*p *= 0.50), sex (*p *= 0.06), Braak NFT stage (*p *= 0.08), and *APOE* ε4 status (*p *= 0.94) – did not differ significantly across groups.

### 3.5 PART with no co‐pathologies

Of the 19 PART participants with no co‐pathologies, seven (37%) had no regions affected, 10 (53%) had one to five regions affected, and only two (11%) had more than five regions affected (Table S1). Across all 19 participants, the most affected regions were the left and right lateral temporal lobes (affected in seven participants each), followed by the left medial temporal lobe (affected in six participants) and right temporal pole (affected in five participants). All other regions were affected in two to four participants. Hypometabolism was scored at a 1 (mild) in all regions except for the left medial temporal lobe, where two participants scored a 2, and the temporal pole, where two participants scored a 2.

### 3.6 Regional visual gradings by Braak NFT stage

**FIGURE 2:**
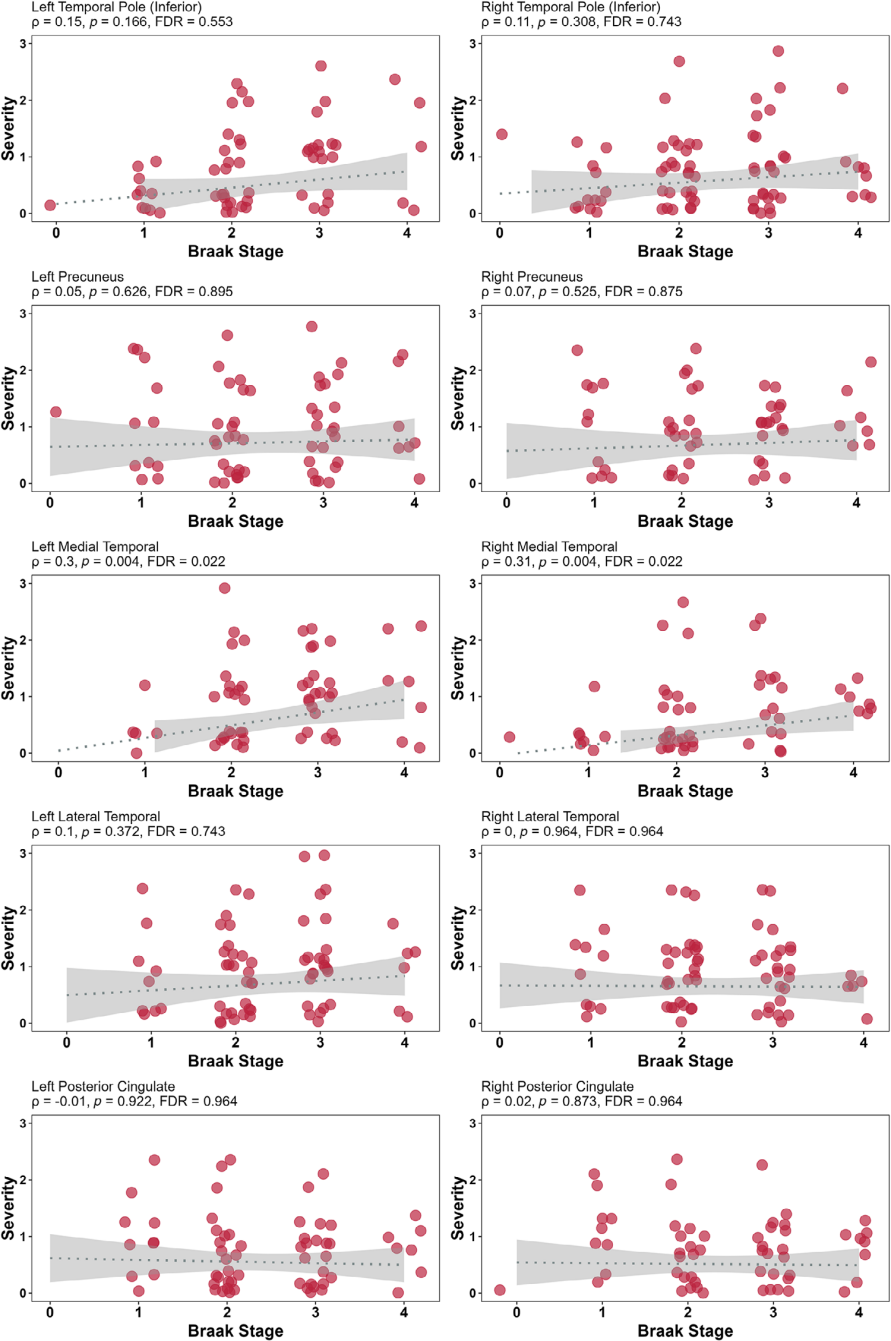
Spearman correlation between severity of each assessed region versus Braak NFT stage (PART + all co‐pathologies). FDR, false discovery rate; NFT, neurofibrillary tangle; PART, primary age‐related tauopathy.

The medial temporal lobe regions demonstrated the strongest associations among regional severity scores and Braak NFT stages (Figure 2). The left medial temporal lobe exhibited a Spearman correlation coefficient (ρ) of 0.30 (*p *= 0.004, FDR‐adjusted *p *= 0.022), while the right medial temporal lobe showed a similar correlation (ρ = 0.31, *p *= 0.004, FDR‐adjusted *p *= 0.022). In contrast, other regions exhibited weak or no correlation with the Braak NFT stage.

### 3.7 Regional SUVRs by co‐pathologies

Linear regression analysis was performed using log‐transformed SUVR values as dependent variables, with TDP‐43, LBD, HpScl, and AGD status as independent variables (Figure S3). Separate models were applied to the left and right medial temporal regions. AGD status was significant for both the left (*p *< 0.001) and right (*p *= 0.018) medial temporal regions, while TDP‐43 status was significant for the left medial temporal region (*p *= 0.012). However, when an interaction term between TDP‐43 and AGD statuses was included in the models, the independent effects of TDP‐43 and AGD were no longer present. Instead, the interaction term became significant (*p *= 0.003 for the left medial temporal lobe and *p *= 0.032 for the right medial temporal lobe), indicating a synergistic interaction between TDP‐43 and AGD statuses. This finding suggests that the combined presence of TDP‐43 and AGD likely drives SUVR variations in the medial temporal regions.

### 3.8 Correlation between SUVR values and visual ratings

To evaluate the relationship between qualitative and quantitative measures of FDG‐PET signal, we assessed the correlation between visual scores and SUVR values in medial temporal regions. Spearman's rank correlation revealed a moderate and statistically significant inverse association in both the left (ρ = −0.50, *p* < 0.001) and right (ρ = −0.55, *p* < 0.001) medial temporal lobes. These findings indicate that higher (worse) visual hypometabolism scores corresponded to lower regional SUVRs, supporting the internal consistency between the two approaches.

### 3.9 Influence of AGD and TDP‐43 in PART

**FIGURE 3:**
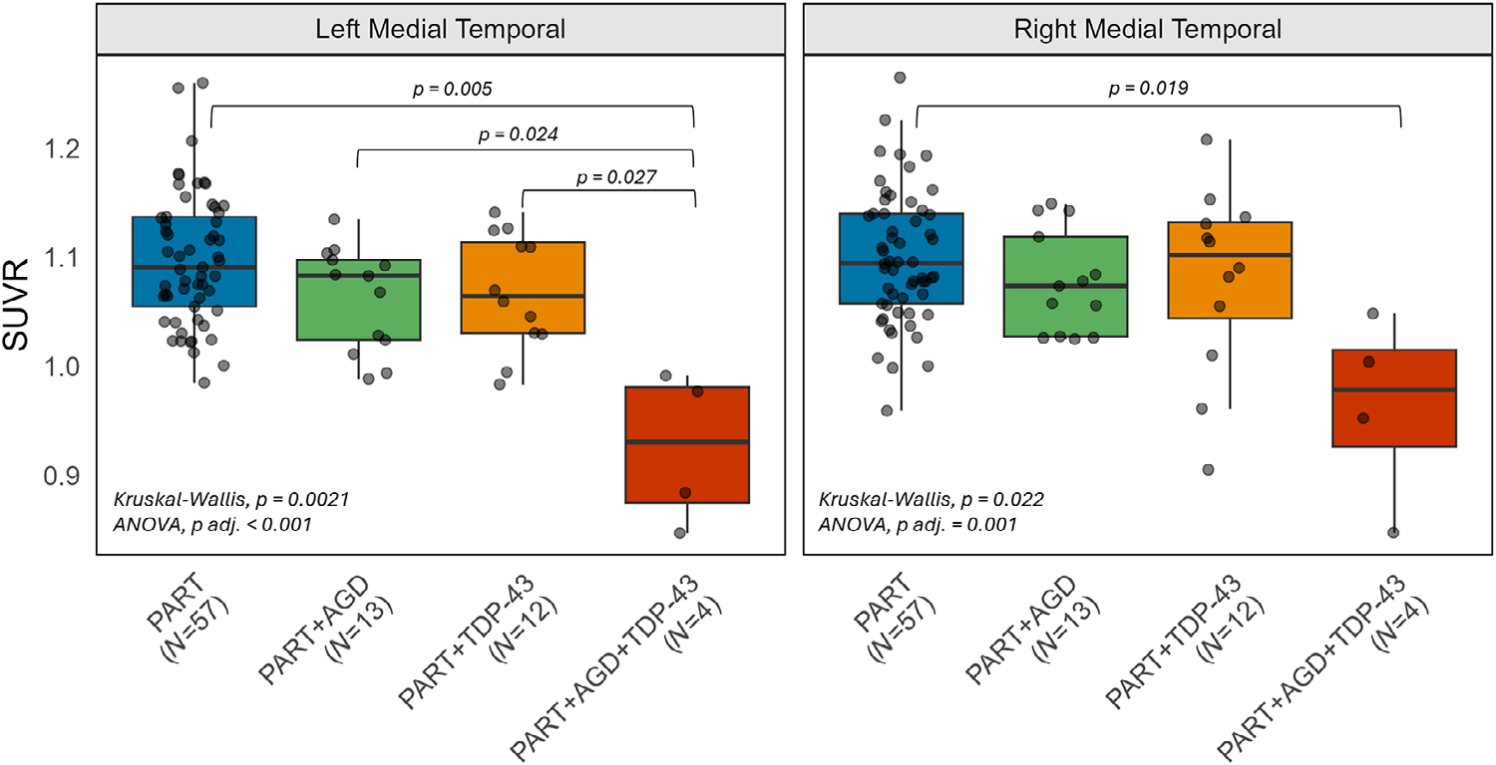
Boxplots showing SUVR values in medial temporal regions across groups: PART, PART+AGD, PART+TDP‐43, and PART+AGD+TDP‐43 (Kruskal–Wallis test and [ANOVA] adjusted for interval between FDG‐PET and death). Post hoc comparisons were performed using the Steel–Dwass test, and only significant (*p* < 0.05) results are displayed. AGD, argyrophilic grain disease; ANOVA, analysis of variance; FDG‐PET, [^18^F]fluorodeoxyglucose positron emission tomography; SUVR, standard uptake value ratio; TDP‐43, TAR DNA‐binding protein 43.

Participants were grouped according to the presence of AGD and TDP‐43 into the following groups: PART (without AGD or TDP‐43), PART+AGD, PART+TDP‐43, and PART+AGD+TDP‐43. Both left (*p *= 0.002) and right (*p *= 0.02) medial temporal SUVRs differed across groups based on Kruskal–Wallis tests and ANOVA adjusted for the interval between FDG‐PET and death (left: *p *< 0.001; right: *p *= 0.001) (Figure 3). On pair‐wise comparisons using Steel–Dwass, left medial temporal lobe SUVR differed between the PART+AGD+TDP‐43 group and the PART (*p *= 0.005), PART+AGD (*p *= 0.024), and PART+TDP‐43 (*p *= 0.027) groups. Right medial temporal lobe SUVR differed between the PART+AGD+TDP‐43 and PART groups (*p *= 0.019), with trends for a difference between the PART+AGD+TDP‐43 and PART+AGD groups (*p *= 0.06).

### 3.10 Part severity (Braak NFT stage) and FDG‐PET hypometabolism

To examine whether increasing PART severity is associated with regional FDG‐PET hypometabolism, we stratified participants by Braak NFT stage (Table S8). We analyzed SUVR values and visual hypometabolism scores across medial and lateral temporal, precuneus, and posterior cingulate regions. In the full cohort, medial temporal SUVR values trended downward with advancing Braak NFT stage, though not significantly (left: *p* = 0.08; right: *p* = 0.42). Similar non‐significant trends were observed in the pure PART subgroup (no co‐pathologies) (left: *p* = 0.40; right: *p* = 0.35). Visual scores also increased modestly with the Braak NFT stage in both groups, particularly in the medial and lateral temporal regions, but the differences were not significant. These results suggest a potential relationship between higher NFT stages and worse regional hypometabolism in PART, even in the absence of co‐pathologies.

## 4 DISCUSSION

PART is defined by the presence of NFTs and minimal absence of Aβ deposition, with diagnosis confirmed only through autopsy. This study provides a detailed analysis of clinical, pathological, and imaging data to investigate the role of FDG‐PET in PART diagnosis and to examine variations in imaging patterns associated with co‐pathologies. Our findings demonstrated that higher Braak NFT stages and an increased number of co‐pathologies correlated with a greater number of brain regions with hypometabolism. Braak NFT stage showed moderate correlations with medial temporal hypometabolism, which agrees with findings in AD, whereby increasing Braak NFT stage is associated with lower FDG‐PET SUVRs in temporoparietal cortical regions. In addition, regional hypometabolism was also significantly associated with co‐pathologies, with medial temporal hypometabolism being associated with AGD, TDP‐43, and HpScl, while parietal hypometabolism was associated with LBD. Hence, medial temporal hypometabolism on FDG‐PET more likely indicates the presence of several pathologies, not just PART.

### 4.1 PART without co‐pathologies

Of our cohort of 88 participants with PART, only 19 had no co‐pathologies. Our findings showed that PART, in the absence of co‐pathologies, was only associated with mild metabolic changes on FDG‐PET, predominantly in the lateral temporal lobes. These changes were subtle, with most visual gradings not exceeding grade 1. Of these participants, 37% showed no regions with hypometabolism. Stratified analysis by Braak NFT stage showed subtle, non‐significant trends toward greater medial temporal hypometabolism with advancing NFT burden, suggesting that PART severity may contribute modestly to metabolic changes even without co‐pathologies. This indicates that PART exhibits an absent or weak metabolic profile in isolation, likely differentiating it metabolically from AD. This may be due to less “damage” from a lower burden of tau deposition in limbic regions, in PART, compared to AD. In fact, the Braak stage was III or lower in these PART‐only participants. Differences in tau conformations or biochemical compositions may also be responsible for the variations observed in PART compared to AD. For example, PART is associated with more ghost tangles (tangles outside of a neuron) than AD.^35^ However, we cannot rule out the possibility that the mild hypometabolism in these patients is spurious and not reflective of underlying tau, mainly since mild hypometabolism was observed in regions that do not have tau pathology at Braak stage III. Further research is warranted to develop reliable methods for the in vivo detection of “pure” PART.

### 4.2 PART with LBD

The presence of LBD significantly alters the FDG‐PET metabolic profile in PART. In our cohort, the co‐occurrence of LBD with PART broadens FDG‐PET hypometabolism beyond the temporal lobes to include parietal regions such as the precuneus and posterior cingulate – patterns that remained significant after FDR correction. These findings are consistent with previous studies^60^ and support the concept of a distinct metabolic footprint of LBD, which reflects the cortical origin and spread of α‐synuclein pathology.^61^, ^62^ These observations highlight the utility of FDG‐PET in identifying the unique metabolic contributions of LBD in the context of PART.

### 4.3 PART with TDP‐43 and AGD

The combination of TDP‐43 and AGD influenced medial temporal metabolism in our PART cohort, although AGD retained significance after correction for multiple comparisons, suggesting that the impact of TDP‐43 may be more variable or secondary to other co‐pathologies. In contrast, AGD showed consistent and robust associations with both left and right medial temporal hypometabolism, supporting its independent contribution. Previous studies^63^, ^64^ separately examined the effects of TDP‐43 and AGD on temporal metabolism, but not their joint effect specifically in PART. Our regression analyses established the interaction between TDP‐43 and AGD as the most statistically significant contributor, underscoring their synergistic relationship. In another study, the co‐occurrence of AGD with TDP‐43 was found to worsen loss of gray matter volume in the temporal lobe beyond that associated with AGD,^65^ suggesting effects on structure, in addition to metabolism, are broader. TDP‐43 proteinopathy has also been linked to unique hypometabolic signatures, such as the medial temporal lobe,^66^, ^67^, ^68^ yet the specificity of these signatures remains unclear.^59^ Likewise, AGD, a pure tauopathy, lacks well‐defined *ante mortem* diagnostic criteria.^69^

### 4.4 PART with HpScl

In our study, HpScl in PART was associated with hypometabolism in the medial temporal and posterior cingulate regions; however, none of these associations remained significant after FDR correction. Previous studies have demonstrated that FDG‐PET reveals distinct metabolic reductions in the hippocampal area, differentiating HpScl from controls and AD.^66^ HpScl is strongly linked to TDP‐43 proteinopathy, also referred to as LATE‐NC by some research groups.^70^ Both TDP‐43 proteinopathy and HpScl predominantly affect the medial temporal lobes, overlapping on imaging, as seen in our study. Mechanisms of LATE‐NC also appear to drive the development of HpScl in aging,^70^ and thus HpScl likely constitutes either a severe manifestation or subset of “pure LATE‐NC.” These point to a close relationship between TDP‐43 proteinopathy and HpScl, such that the latter is likely to develop as a downstream effect of TDP‐43‐mediated pathological and metabolic failure.^59^

### 4.5 Strengths and limitations

Our study had several strengths. First, we had a large cohort of autopsy‐diagnosed PART participants who had an *ante mortem* FDG‐PET scan. Second, all pathological diagnoses were completed by a board‐certified neuropathologist with expertise in the diagnosis of neurodegenerative diseases. Limitations include a small sample of isolated PART cases, with most participants having co‐pathologies, and the fact that the time between FDG‐PET and death varied, suggesting a potentially higher burden and/or disease stage at the time of autopsy. Furthermore, tau burden was not measured, and Braak NFT staging does not account for regional heterogeneity in pathology. This limitation affects the stratification of PART severity and indicates a gap in knowledge regarding the role of ghost tangles in disease severity. More studies are needed to determine if tau burden, ghost tangles, or both drive PART severity. We also did not compare “definite” PART (Thal phase 0) with “probable” PART (Thal phases 1 and 2) due to small group sizes, though such an analysis may help clarify whether sparse Aβ deposition affects FDG‐PET patterns. Finally, visual scores and SUVR values may not fully align, as visual ratings were influenced by focal hypometabolism within a region, while SUVRs reflected median uptake across the full anatomical area.

## 5 CONCLUSIONS

Our study highlights the need to examine co‐pathologies, especially AGD and TDP‐43, and their respective roles in mediating regional metabolic and pathological alterations in PART. These results also highlight the medial temporal regions as sites of key pathological interactions and metabolic change and thus underscore their clinical and research relevance in neurodegeneration. PART alone appears to have minimal effect on medial temporal lobe hypometabolism. While FDG‐PET is not currently part of routine diagnostic practice for PART, our findings support its potential value as a research tool to probe underlying neuropathology in vivo. Future studies should further explore the mechanisms underlying these synergistic effects and their implications for disease progression and treatment strategies.

## CONFLICT OF INTEREST STATEMENT

Anna Lavrova, Nha Trang Thu Pham, Cynthia J. Vernon, Jonathan Graff‐Radford, Bradley F. Boeve, David S. Knopman, Aivi Nguyen, and Reichard R. Ross report no conflicts of interest. Dennis W. Dickson, Jennifer L. Whitwell, and Keith A. Josephs receive research support from the National Institutes of Health (NIH) (National Institute of Aging/National Institute of Neurological Disorders and Stroke/National Institute on Deafness and Other Communication Disorders). Val J. Lowe consults for Bayer Schering Pharma, Piramal Life Sciences, Life Molecular Imaging, Eisai Inc., AVID Radiopharmaceuticals, and Merck Research and receives research support from GE Healthcare, Siemens Molecular Imaging, AVID Radiopharmaceuticals, and the NIH (NIA, National Cancer Institute). Kejal Kantarci received research support from NIH, Alzheimer's Drug Discovery Foundation, and Eli Lilly. She consulted Biogen with no personal compensation. She is supported by the Katherine B. Andersen Endowed Professorship.

Ronald C. Petersen serves on data monitoring committees for Janssen Alzheimer Immunotherapy and is a consultant for Biogen, Roche, Merck, and Genentech, Inc.; receives publishing royalties from Mild Cognitive Impairment (Oxford University Press, 2003) and receives research support from the NIH/NIA. Clifford R. Jack, Jr., receives no personal or institutional compensation from any commercial entity. He receives research support from NIH, the GHR Foundation, and the Alexander Family Alzheimer's Disease Research Professorship of the Mayo Clinic. Author disclosures are available in the Supporting Information.

## CONSENT STATEMENT

This study was approved by the Mayo Clinic Institutional Review Board, and all patients or their proxies signed a written informed consent form before taking part in any research activities in accordance with the Declaration of Helsinki.

## Supporting information



Supporting Information

Supporting Information
