# Supplementary material for: 18F‐FDG PET in detection of primary age‐related tauopathy (PART) – Is there a role? Insights from an imaging‐pathology correlation study
Source: Alzheimers Dement. 2025 Aug 19;21(8):e70568. doi: 10.1002/alz.70568 (PMC12361867; doi:10.1002/alz.70568)

**Supplemental Table 1**. Demographic and clinical features by the number of affected regions.

|  | | **N of affected regions** | | |  | |
| --- | --- | --- | --- | --- | --- | --- |
|  | None (N=15) | | From 1 to 5 (N=37) | More than 5 (N=34) | | *p-value* |
| **Age at death** |  | |  |  | | 0.58 (1) |
| Median (Q1, Q3) | 83.4 (79.3, 91.2) | | 87.7 (81.7, 92.0) | 86.2 (76.7, 92.0) | |  |
| **Sex** |  | |  |  | | 0.87 (2) |
| F | 6 (40.0%) | | 13 (35.1%) | 11 (32.4%) | |  |
| M | 9 (60.0%) | | 24 (64.9%) | 23 (67.6%) | |  |
| **Braak stage** |  | |  |  | | 0.01 (2) |
| 0 | 0 (0.0%) | | 1 (2.7%) | 0 (0.0%) | |  |
| I | 6 (40.0%) | | 2 (5.4%) | 5 (14.7%) | |  |
| II | 8 (53.3%) | | 15 (40.5%) | 10 (29.4%) | |  |
| III | 1 (6.7%) | | 16 (43.2%) | 13 (38.2%) | |  |
| IV | 0 (0.0%) | | 3 (8.1%) | 6 (17.6%) | |  |
| **Thal phase** |  | |  |  | | 0.35 (2) |
| 0 | 10 (66.7%) | | 16 (43.2%) | 14 (41.2%) | |  |
| 1 | 4 (26.7%) | | 11 (29.7%) | 9 (26.5%) | |  |
| 2 | 1 (6.7%) | | 10 (27.0%) | 11 (32.4%) | |  |
| **CERAD** |  | |  |  | | 0.37 (2) |
| Normal | 13 (86.7%) | | 20 (54.1%) | 20 (58.8%) | |  |
| Mild | 2 (13.3%) | | 10 (27.0%) | 9 (26.5%) | |  |
| Moderate | 0 (0.0%) | | 6 (16.2%) | 5 (14.7%) | |  |
| Frequent | 0 (0.0%) | | 1 (2.7%) | 0 (0.0%) | |  |
| **Age at FDG** |  | |  |  | | 0.51 (1) |
| Median (Q1, Q3) | 77.4 (73.6, 87.9) | | 81.9 (78.2, 87.0) | 80.6 (74.5, 87.4) | |  |
| **Time between FDG and death (y)** |  | |  |  | | 0.94 (1) |
| Median (Q1, Q3) | 4.1 (1.5, 8.1) | | 4.0 (3.2, 7.0) | 3.8 (2.5, 7.4) | |  |
| **MMSE** **at FDG** |  | |  |  | | < 0.01 (1) |
| Median (Q1, Q3) | 28.0 (27.0, 29.0) | | 29.0 (27.0, 29.0) | 26.0 (23.0, 28.0) | |  |
| **Dementia (clinical)** |  | |  |  | | 0.07 (2) |
| no | 10 (66.7%) | | 27 (73.0%) | 16 (47.1%) | |  |
| yes | 5 (33.3%) | | 10 (27.0%) | 18 (52.9%) | |  |
| **Clinical diagnosis** |  | |  |  | | 0.03 (2) |
| Atypical Alzheimer’s dementia | 0 (0.0%) | | 0 (0.0%) | 2 (5.9%) | |  |
| Typical Alzheimer’s dementia | 0 (0.0%) | | 2 (5.4%) | 2 (5.9%) | |  |
| Dementia – Unclassifiable / Mokri syndrome | 1 (6.7%) | | 0 (0.0%) | 1 (2.9%) | |  |
| Dementia with Lewy Bodies | 0 (0.0%) | | 2 (5.4%) | 10 (29.4%) | |  |
| Mild Cognitive Impairment | 4 (26.7%) | | 6 (16.2%) | 3 (8.8%) | |  |
| Cognitively Normal | 10 (66.7%) | | 27 (73.0%) | 16 (47.1%) | |  |
| ***APOE*** |  | |  |  | | 0.22 (2) |
| negative | 14 (100.0%) | | 30 (81.1%) | 28 (82.4%) | |  |
| positive | 0 (0.0%) | | 7 (18.9%) | 6 (17.6%) | |  |
| **TDP-43 status** |  | |  |  | | 0.32 (2) |
| negative | 13 (86.7%) | | 32 (86.5%) | 25 (73.5%) | |  |
| positive | 2 (13.3%) | | 5 (13.5%) | 9 (26.5%) | |  |
| **HpScl status** |  | |  |  | | 0.51 (2) |
| negative | 14 (93.3%) | | 33 (89.2%) | 28 (82.4%) | |  |
| positive | 1 (6.7%) | | 4 (10.8%) | 6 (17.6%) | |  |
| **ARTAG status** |  | |  |  | | 0.42 (2) |
| negative | 10 (71.4%) | | 23 (62.2%) | 26 (76.5%) | |  |
| positive | 4 (28.6%) | | 14 (37.8%) | 8 (23.5%) | |  |
| **AGD status** |  | |  |  | | 0.11 (2) |
| negative | 15 (100.0%) | | 28 (75.7%) | 26 (76.5%) | |  |
| positive | 0 (0.0%) | | 9 (24.3%) | 8 (23.5%) | |  |
| **LBD status** |  | |  |  | | < 0.01 (2) |
| negative | 15 (100.0%) | | 26 (70.3%) | 13 (38.2%) | |  |
| positive | 0 (0.0%) | | 11 (29.7%) | 21 (61.8%) | |  |
| **LBD stage** |  | |  |  | | < 0.01 (2) |
| Amygdala predominant | 0 (0.0%) | | 6 (16.2%) | 4 (11.8%) | |  |
| Brainstem predominant | 0 (0.0%) | | 3 (8.1%) | 7 (20.6%) | |  |
| Diffuse/neocortical | 0 (0.0%) | | 2 (5.4%) | 9 (26.5%) | |  |
| Limbic/transitional | 0 (0.0%) | | 0 (0.0%) | 1 (2.9%) | |  |
| None | 15 (100.0%) | | 26 (70.3%) | 13 (38.2%) | |  |
| **PART ± co-pathology [TDP-43, AGD, LBD, HpScl]** |  | |  |  | | < 0.01 (2) |
| PART only (N=19) | 7 (46.7%) | | 10 (27.0%) | 2 (5.9%) | |  |
| PART + co-pathology (N=69) | 8 (53.3%) | | 27 (73.0%) | 32 (94.1%) | |  |

*Note:* (1) Kruskal-Wallis rank sum test, (2) Pearson's Chi-squared test. Data are shown as N (%) or median (Q1, Q3).

*Abbreviations:* AGD = argyrophilic grain disease, *APOE* = apolipoprotein, ARTAG = age‐related tau astrogliopathy, CERAD = Consortium to Establish a Registry for Alzheimer's Disease, , , FDG-PET = positron emission tomography with ^18^F-fluorodeoxyglucose, HpScl = hippocampal sclerosis, LBD = Lewy body disease, , MCI = Mild Cognitive Impairment, MMSE = Mini-Mental State Examination, PART = Primary Age-Related Tauopathy, , TDP-43 = trans-active response DNA-binding protein of 43 kDa.

**Supplemental Table 2.** Ordinal Logistic Regression: Braak NFT Stage.

| Predictor | OR | Std. Error | z-value | 95% CI (Lower–Upper) | p-value |
| --- | --- | --- | --- | --- | --- |
| From 1 to 5 regions affected | 6.23 | 0.594 | 3.08 | 2.00–20.79 | 0.002 |
| More than 5 regions affected | 7.83 | 0.614 | 3.35 | 2.42–27.21 | <0.001 |
| Age at FDG-PET | 1.03 | 0.024 | 1.15 | 0.98–1.08 | 0.252 |

**Supplemental Table 3.** Linear Regression: MMSE.

| Predictor | Estimate | Std. Error | t-value | 95% CI (Lower–Upper) | p-value |
| --- | --- | --- | --- | --- | --- |
| From 1 to 5 regions affected | -0.66 | 1.14 | -0.58 | -2.93–1.60 | 0.562 |
| More than 5 regions affected | -2.87 | 1.15 | -2.49 | -5.16–0.58 | 0.015 |
| Age at FDG-PET | 0.096 | 0.049 | 1.94 | -0.002–0.194 | 0.055 |

**Supplemental Table 4.** Multinomial Logistic Regression: Dementia Type.

| Group | Predictor | OR | Std. Error | z-value | 95% CI (Lower–Upper) | p-value |
| --- | --- | --- | --- | --- | --- | --- |
| DLB | From 1 to 5 regions affected | 144.24 | 2.19 | 2.27 | 1.98–10,516.91 | 0.023 |
|  | More than 5 regions affected | 701.34 | 2.15 | 3.04 | 10.31–47,693.61 | 0.002 |
|  | Age at FDG-PET | 0.92 | 0.08 | -1.08 | 0.79–1.07 | 0.281 |
| None | From 1 to 5 regions affected | 0.004 | 2.13 | -2.55 | <0.001–0.28 | 0.011 |
|  | More than 5 regions affected | 0.003 | 2.10 | -2.83 | <0.001–0.16 | 0.005 |
|  | Age at FDG-PET | 1.001 | 0.072 | 0.01 | 0.87–1.15 | 0.993 |
| Other | From 1 to 5 regions affected | 0.002 | 2.16 | -2.88 | <0.001–0.14 | 0.004 |
|  | More than 5 regions affected | 0.002 | 2.12 | -2.93 | <0.001–0.13 | 0.003 |
|  | Age at FDG-PET | 0.99 | 0.076 | -0.18 | 0.85–1.15 | 0.856 |

**Supplemental Table 5.** Firth Logistic Regression: LBD Status.

| Predictor | OR | Std. Error | z-value | 95% CI (Lower–Upper) | p-value |
| --- | --- | --- | --- | --- | --- |
| From 1 to 5 regions affected | 13.70 | 1.46 | 1.79 | 0.46–7.50 | 0.012 |
| More than 5 regions affected | 48.25 | 1.46 | 2.66 | 1.73–8.76 | <0.001 |
| Age at FDG-PET | 0.98 | 0.03 | -0.57 | -0.08–0.04 | 0.579 |

**Supplemental Table 6.** Ordinal Logistic Regression: LBD Stage.

| Predictor | OR | Std. Error | t-value | 95% CI Lower | 95% CI Upper | p-value |
| --- | --- | --- | --- | --- | --- | --- |
| From 1 to 5 regions affected | 1.55×10⁻⁷ | 0.89 | -17.53 | 2.68×10⁻⁸ | 8.95×10⁻⁷ | <0.001 |
| More than 5 regions affected | 4.06×10⁻⁸ | 0.81 | -20.89 | 8.22×10⁻⁹ | 2.00×10⁻⁷ | <0.001 |
| Age at FDG-PET | 1.01 | 0.03 | 0.19 | 0.95 | 1.06 | 0.845 |

**Supplemental Table 7.** Demographic and clinical features by the number of co-pathologies.

|  | | | **N of co-pathologies** | | | |  | | |
| --- | --- | --- | --- | --- | --- | --- | --- | --- | --- |
|  | 0 (N=26) | 1 (N=34) | | 2 (N=15) | 3 (N=7) | 4 (N=3) | | 5 (N=1) | *p-value* |
| **Age at death** |  |  | |  |  |  | |  | 0.50 (1) |
| Median (Q1, Q3) | 83.6 (77.1, 91.9) | 83.5 (79.5, 91.1) | | 88.2 (84.2, 94.2) | 87.3 (86.0, 91.6) | 88.8 (87.7, 90.0) | | 95.5 (95.5, 95.5) |  |
| **Sex** |  |  | |  |  |  | |  | 0.06 (2) |
| F | 9 (34.6%) | 10 (29.4%) | | 10 (66.7%) | 1 (14.3%) | 0 (0.0%) | | 0 (0.0%) |  |
| M | 17 (65.4%) | 24 (70.6%) | | 5 (33.3%) | 6 (85.7%) | 3 (100.0%) | | 1 (100.0%) |  |
| **Braak stage** |  |  | |  |  |  | |  | 0.08 (2) |
| 0 | 0 (0.0%) | 0 (0.0%) | | 0 (0.0%) | 1 (14.3%) | 0 (0.0%) | | 0 (0.0%) |  |
| I | 3 (11.5%) | 8 (23.5%) | | 2 (13.3%) | 0 (0.0%) | 0 (0.0%) | | 0 (0.0%) |  |
| II | 15 (57.7%) | 10 (29.4%) | | 3 (20.0%) | 2 (28.6%) | 2 (66.7%) | | 1 (100.0%) |  |
| III | 8 (30.8%) | 11 (32.4%) | | 8 (53.3%) | 2 (28.6%) | 1 (33.3%) | | 0 (0.0%) |  |
| IV | 0 (0.0%) | 5 (14.7%) | | 2 (13.3%) | 2 (28.6%) | 0 (0.0%) | | 0 (0.0%) |  |
| **Thal phase** |  |  | |  |  |  | |  | 0.82 (2) |
| 0 | 11 (42.3%) | 16 (47.1%) | | 8 (53.3%) | 2 (28.6%) | 2 (66.7%) | | 1 (100.0%) |  |
| 1 | 8 (30.8%) | 7 (20.6%) | | 5 (33.3%) | 3 (42.9%) | 1 (33.3%) | | 0 (0.0%) |  |
| 2 | 7 (26.9%) | 11 (32.4%) | | 2 (13.3%) | 2 (28.6%) | 0 (0.0%) | | 0 (0.0%) |  |
| **CERAD** |  |  | |  |  |  | |  | 0.05 (2) |
| Absent | 14 (53.8%) | 15 (44.1%) | | 7 (46.7%) | 3 (42.9%) | 0 (0.0%) | | 0 (0.0%) |  |
| Frequent | 0 (0.0%) | 0 (0.0%) | | 0 (0.0%) | 1 (14.3%) | 0 (0.0%) | | 0 (0.0%) |  |
| Mild | 7 (26.9%) | 9 (26.5%) | | 1 (6.7%) | 3 (42.9%) | 1 (33.3%) | | 0 (0.0%) |  |
| Moderate | 3 (11.5%) | 5 (14.7%) | | 3 (20.0%) | 0 (0.0%) | 0 (0.0%) | | 0 (0.0%) |  |
| Normal | 2 (7.7%) | 5 (14.7%) | | 4 (26.7%) | 0 (0.0%) | 2 (66.7%) | | 1 (100.0%) |  |
| **Age at FDG-PET** |  |  | |  |  |  | |  | 0.42 (1) |
| Median (Q1, Q3) | 81.2 (73.6, 87.4) | 80.7 (74.3, 84.4) | | 80.8 (77.5, 87.7) | 84.2 (83.3, 87.0) | 80.2 (78.5, 81.0) | | 90.3 (90.3, 90.3) |  |
| **Time between FDG-PET and death (y)** |  |  | |  |  |  | |  | 0.07 (1) |
| Median (Q1, Q3) | 3.3 (1.8, 5.4) | 3.5 (2.2, 7.2) | | 6.0 (4.0, 7.8) | 4.0 (2.7, 4.1) | 9.7 (8.3, 10.4) | | 5.2 (5.2, 5.2) |  |
| **MMSE** **at FDG-PET** |  |  | |  |  |  | |  | 0.04 (1) |
| Median (Q1, Q3) | 28.0 (27.0, 29.0) | 27.0 (22.2, 29.0) | | 28.5 (26.5, 29.0) | 28.0 (25.0, 28.5) | 26.0 (23.0, 27.5) | | 23.0 (23.0, 23.0) |  |
| **Dementia (clinical)** |  |  | |  |  |  | |  | 0.33 (2) |
| no | 20 (76.9%) | 19 (55.9%) | | 9 (60.0%) | 4 (57.1%) | 1 (33.3%) | | 0 (0.0%) |  |
| yes | 6 (23.1%) | 15 (44.1%) | | 6 (40.0%) | 3 (42.9%) | 2 (66.7%) | | 1 (100.0%) |  |
| **Clinical diagnosis** |  |  | |  |  |  | |  | < 0.01 (2) |
| Atypical Alzheimer’s dementia | 0 (0.0%) | 2 (5.9%) | | 0 (0.0%) | 0 (0.0%) | 0 (0.0%) | | 0 (0.0%) |  |
| Typical Alzheimer’s dementia | 0 (0.0%) | 0 (0.0%) | | 0 (0.0%) | 3 (42.9%) | 1 (33.3%) | | 0 (0.0%) |  |
| Dementia – Unclassifiable / Mokri syndrome | 1 (3.8%) | 1 (2.9%) | | 0 (0.0%) | 0 (0.0%) | 0 (0.0%) | | 0 (0.0%) |  |
| Dementia with Lewy Bodies | 0 (0.0%) | 8 (23.5%) | | 3 (20.0%) | 0 (0.0%) | 0 (0.0%) | | 1 (100.0%) |  |
| Mild Cognitive Impairment | 5 (19.2%) | 4 (11.8%) | | 3 (20.0%) | 0 (0.0%) | 1 (33.3%) | | 0 (0.0%) |  |
| Cognitively Normal | 20 (76.9%) | 19 (55.9%) | | 9 (60.0%) | 4 (57.1%) | 1 (33.3%) | | 0 (0.0%) |  |
| ***APOE*** |  |  | |  |  |  | |  | 0.94 (2) |
| negative | 20 (80.0%) | 29 (85.3%) | | 13 (86.7%) | 6 (85.7%) | 3 (100.0%) | | 1 (100.0%) |  |
| positive | 5 (20.0%) | 5 (14.7%) | | 2 (13.3%) | 1 (14.3%) | 0 (0.0%) | | 0 (0.0%) |  |

*Note:* (1) Kruskal-Wallis rank sum test, (2) Pearson's Chi-squared test. Data are shown as N (%) or median (Q1, Q3).

*Abbreviations:* AGD = argyrophilic grain disease, *APOE* = apolipoprotein, ARTAG = age‐related tau astrogliopathy, CERAD = Consortium to Establish a Registry for Alzheimer's Disease, , FDG-PET = positron emission tomography with ^18^F-fluorodeoxyglucose, HpScl = hippocampal sclerosis, LBD = Lewy body disease, , MCI = Mild Cognitive Impairment, MMSE = Mini-Mental State Examination, PART = Primary Age Related Tauopathy, TDP-43 = trans-active response DNA-binding protein of 43 kDa.

**Supplemental Table 8.** Regional FDG-PET SUVR values and visual hypometabolism scores by Braak NFT stage in the full cohort and in pure PART cases.

| Full cohort (PART + co-pathologies) | | | | | | | Pure PART (no co-pathologies) | | | |
| --- | --- | --- | --- | --- | --- | --- | --- | --- | --- | --- |
|  | Braak 0 (N=1) | Braak I (N=13) | Braak II (N=33) | Braak III (N=30) | Braak IV (N=9) | *p-value* | Braak I (N=3) | Braak II (N=9) | Braak III (N=7) | *p-value* |
| SUVR (Medial Temporal Left) | 1.06 (1.06, 1.06) | 1.13 (1.10, 1.17) | 1.10 (1.02, 1.14) | 1.07 (1.03, 1.10) | 1.07 (1.04, 1.08) | 0.08 (1) | 1.11 (1.05, 1.14) | 1.10 (1.06, 1.12) | 1.07 (1.03, 1.08) | 0.40 (1) |
| SUVR (Medial Temporal Right) | 1.08 (1.08, 1.08) | 1.12 (1.06, 1.16) | 1.09 (1.05, 1.14) | 1.08 (1.05, 1.11) | 1.07 (1.06, 1.11) | 0.42 (1) | 1.11 (1.04, 1.15) | 1.10 (1.08, 1.13) | 1.08 (1.04, 1.09) | 0.35 (1) |
| Temporal pole Left |  |  |  |  |  | 0.62 (2) |  |  |  | 0.62 (2) |
| 0 | 1 (100.0%) | 10 (76.9%) | 21 (63.6%) | 16 (53.3%) | 6 (66.7%) |  | 3 (100.0%) | 7 (77.8%) | 5 (71.4%) |  |
| 1 | 0 (0.0%) | 3 (23.1%) | 8 (24.2%) | 10 (33.3%) | 1 (11.1%) |  | 0 (0.0%) | 1 (11.1%) | 2 (28.6%) |  |
| 2 | 0 (0.0%) | 0 (0.0%) | 4 (12.1%) | 2 (6.7%) | 2 (22.2%) |  | 0 (0.0%) | 1 (11.1%) | 0 (0.0%) |  |
| 3 | 0 (0.0%) | 0 (0.0%) | 0 (0.0%) | 2 (6.7%) | 0 (0.0%) |  |  |  |  |  |
| Temporal pole Right |  |  |  |  |  | 0.81 (2) |  |  |  | 0.14 (2) |
| 0 | 0 (0.0%) | 9 (69.2%) | 17 (51.5%) | 16 (53.3%) | 4 (44.4%) |  | 3 (100.0%) | 5 (55.6%) | 6 (85.7%) |  |
| 1 | 1 (100.0%) | 4 (30.8%) | 14 (42.4%) | 9 (30.0%) | 4 (44.4%) |  | 0 (0.0%) | 4 (44.4%) | 0 (0.0%) |  |
| 2 | 0 (0.0%) | 0 (0.0%) | 1 (3.0%) | 4 (13.3%) | 1 (11.1%) |  | 0 (0.0%) | 0 (0.0%) | 1 (14.3%) |  |
| 3 | 0 (0.0%) | 0 (0.0%) | 1 (3.0%) | 1 (3.3%) | 0 (0.0%) |  |  |  |  |  |
| Precuneus Left |  |  |  |  |  | 0.83 (2) |  |  |  | 0.59 (2) |
| 0 | 0 (0.0%) | 7 (53.8%) | 19 (57.6%) | 15 (50.0%) | 3 (33.3%) |  | 3 (100.0%) | 7 (77.8%) | 5 (71.4%) |  |
| 1 | 1 (100.0%) | 2 (15.4%) | 8 (24.2%) | 9 (30.0%) | 4 (44.4%) |  | 0 (0.0%) | 2 (22.2%) | 2 (28.6%) |  |
| 2 | 0 (0.0%) | 4 (30.8%) | 5 (15.2%) | 5 (16.7%) | 2 (22.2%) |  |  |  |  |  |
| 3 | 0 (0.0%) | 0 (0.0%) | 1 (3.0%) | 1 (3.3%) | 0 (0.0%) |  |  |  |  |  |
| Precuneus Right |  |  |  |  |  | 0.59 (2) |  |  |  | 0.65 (2) |
| 0 | 1 (100.0%) | 7 (53.8%) | 18 (54.5%) | 16 (53.3%) | 2 (22.2%) |  | 3 (100.0%) | 7 (77.8%) | 6 (85.7%) |  |
| 1 | 0 (0.0%) | 2 (15.4%) | 9 (27.3%) | 11 (36.7%) | 5 (55.6%) |  | 0 (0.0%) | 2 (22.2%) | 1 (14.3%) |  |
| 2 | 0 (0.0%) | 4 (30.8%) | 5 (15.2%) | 2 (6.7%) | 2 (22.2%) |  |  |  |  |  |
| 3 | 0 (0.0%) | 0 (0.0%) | 1 (3.0%) | 1 (3.3%) | 0 (0.0%) |  |  |  |  |  |
| Medial Temporal Left |  |  |  |  |  | 0.25 (2) |  |  |  | 0.07 (2) |
| 0 | 1 (100.0%) | 12 (92.3%) | 20 (60.6%) | 13 (43.3%) | 4 (44.4%) |  | 3 (100.0%) | 5 (55.6%) | 5 (71.4%) |  |
| 1 | 0 (0.0%) | 1 (7.7%) | 8 (24.2%) | 12 (40.0%) | 3 (33.3%) |  | 0 (0.0%) | 4 (44.4%) | 0 (0.0%) |  |
| 2 | 0 (0.0%) | 0 (0.0%) | 3 (9.1%) | 5 (16.7%) | 2 (22.2%) |  | 0 (0.0%) | 0 (0.0%) | 2 (28.6%) |  |
| 3 | 0 (0.0%) | 0 (0.0%) | 2 (6.1%) | 0 (0.0%) | 0 (0.0%) |  |  |  |  |  |
| Medial Temporal Right |  |  |  |  |  | 0.08 (2) |  |  |  | 0.29 (2) |
| 0 | 1 (100.0%) | 12 (92.3%) | 24 (72.7%) | 20 (66.7%) | 2 (22.2%) |  | 3 (100.0%) | 7 (77.8%) | 7 (100.0%) |  |
| 1 | 0 (0.0%) | 1 (7.7%) | 6 (18.2%) | 8 (26.7%) | 7 (77.8%) |  | 0 (0.0%) | 2 (22.2%) | 0 (0.0%) |  |
| 2 | 0 (0.0%) | 0 (0.0%) | 2 (6.1%) | 2 (6.7%) | 0 (0.0%) |  |  |  |  |  |
| 3 | 0 (0.0%) | 0 (0.0%) | 1 (3.0%) | 0 (0.0%) | 0 (0.0%) |  |  |  |  |  |
| Lateral Temporal Left |  |  |  |  |  | 0.88 (2) |  |  |  | 0.35 (2) |
| 0 | 1 (100.0%) | 8 (61.5%) | 16 (48.5%) | 12 (40.0%) | 5 (55.6%) |  | 3 (100.0%) | 5 (55.6%) | 4 (57.1%) |  |
| 1 | 0 (0.0%) | 3 (23.1%) | 12 (36.4%) | 12 (40.0%) | 3 (33.3%) |  | 0 (0.0%) | 4 (44.4%) | 3 (42.9%) |  |
| 2 | 0 (0.0%) | 2 (15.4%) | 5 (15.2%) | 4 (13.3%) | 1 (11.1%) |  |  |  |  |  |
| 3 | 0 (0.0%) | 0 (0.0%) | 0 (0.0%) | 2 (6.7%) | 0 (0.0%) |  |  |  |  |  |
| Lateral Temporal Right |  |  |  |  |  | 0.82 (2) |  |  |  | 0.35 (2) |
| 0 | 1 (100.0%) | 7 (53.8%) | 13 (39.4%) | 13 (43.3%) | 5 (55.6%) |  | 3 (100.0%) | 5 (55.6%) | 4 (57.1%) |  |
| 1 | 0 (0.0%) | 4 (30.8%) | 17 (51.5%) | 13 (43.3%) | 4 (44.4%) |  | 0 (0.0%) | 4 (44.4%) | 3 (42.9%) |  |
| 2 | 0 (0.0%) | 2 (15.4%) | 3 (9.1%) | 4 (13.3%) | 0 (0.0%) |  |  |  |  |  |
| Posterior cingulate Left |  |  |  |  |  | 0.90 (2) |  |  |  | 0.65 (2) |
| 0 | 1 (100.0%) | 6 (46.2%) | 20 (60.6%) | 17 (56.7%) | 4 (44.4%) |  | 2 (66.7%) | 8 (88.9%) | 6 (85.7%) |  |
| 1 | 0 (0.0%) | 5 (38.5%) | 9 (27.3%) | 11 (36.7%) | 5 (55.6%) |  | 1 (33.3%) | 1 (11.1%) | 1 (14.3%) |  |
| 2 | 0 (0.0%) | 2 (15.4%) | 3 (9.1%) | 2 (6.7%) | 0 (0.0%) |  |  |  |  |  |
| 3 | 0 (0.0%) | 0 (0.0%) | 1 (3.0%) | 0 (0.0%) | 0 (0.0%) |  |  |  |  |  |
| Posterior cingulate Right |  |  |  |  |  | 0.64 (2) |  |  |  | 0.29 (2) |
| 0 | 1 (100.0%) | 6 (46.2%) | 21 (63.6%) | 18 (60.0%) | 3 (33.3%) |  | 2 (66.7%) | 8 (88.9%) | 7 (100.0%) |  |
| 1 | 0 (0.0%) | 5 (38.5%) | 9 (27.3%) | 11 (36.7%) | 6 (66.7%) |  | 1 (33.3%) | 1 (11.1%) | 0 (0.0%) |  |
| 2 | 0 (0.0%) | 2 (15.4%) | 2 (6.1%) | 1 (3.3%) | 0 (0.0%) |  |  |  |  |  |
| 3 | 0 (0.0%) | 0 (0.0%) | 1 (3.0%) | 0 (0.0%) | 0 (0.0%) |  |  |  |  |  |

*Note:* (1) Kruskal-Wallis rank sum test, (2) Pearson's Chi-squared test. Data are shown as N (%) or median (Q1, Q3).

**Supplemental Figure 1.** Different patterns of FDG hypometabolism in different pathologies.


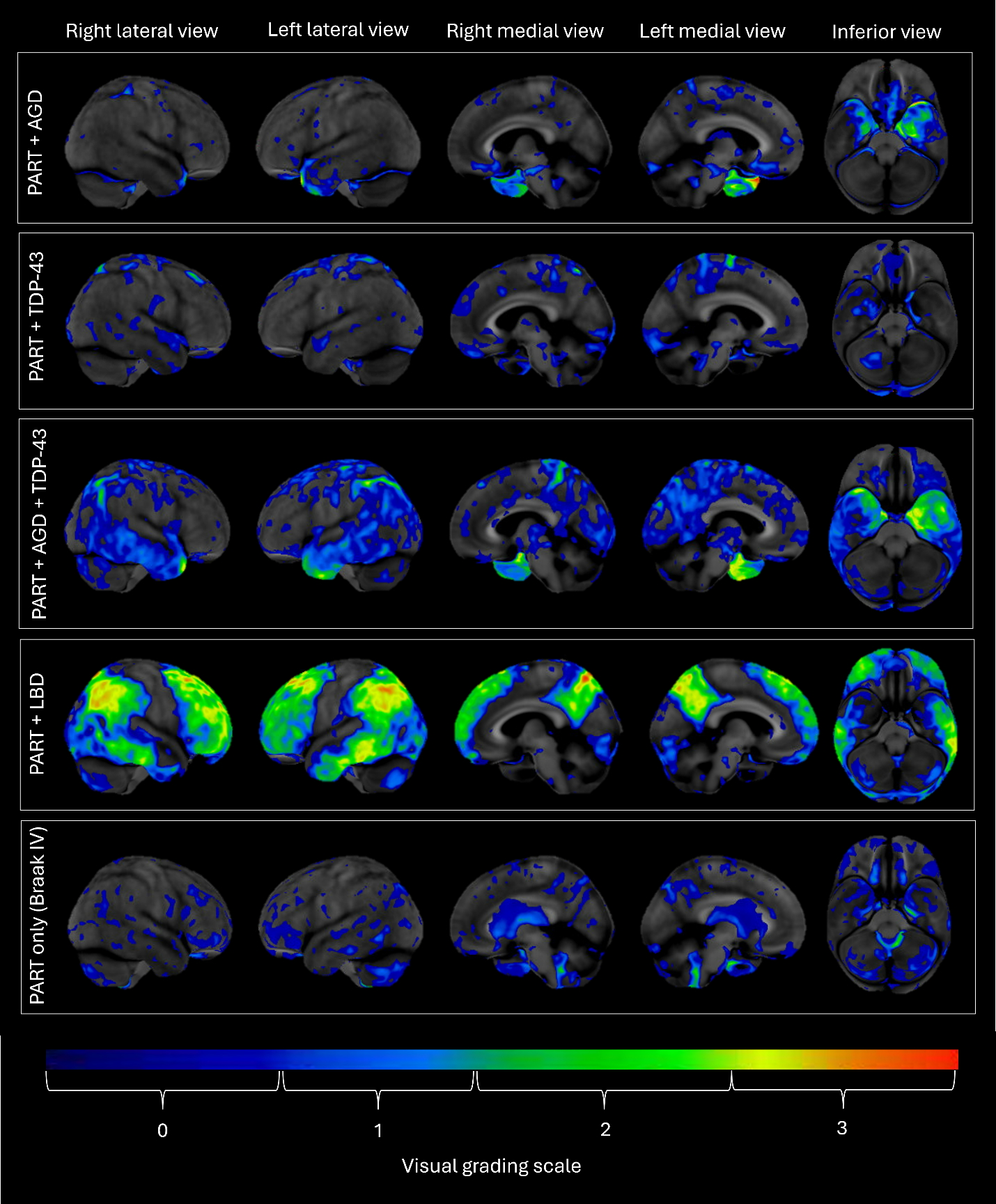


**Supplemental Figure 2.** Correlation between the number of affected regions and the number of co-pathologies. A moderate positive correlation is observed between the number of affected regions and the number of co-pathologies.


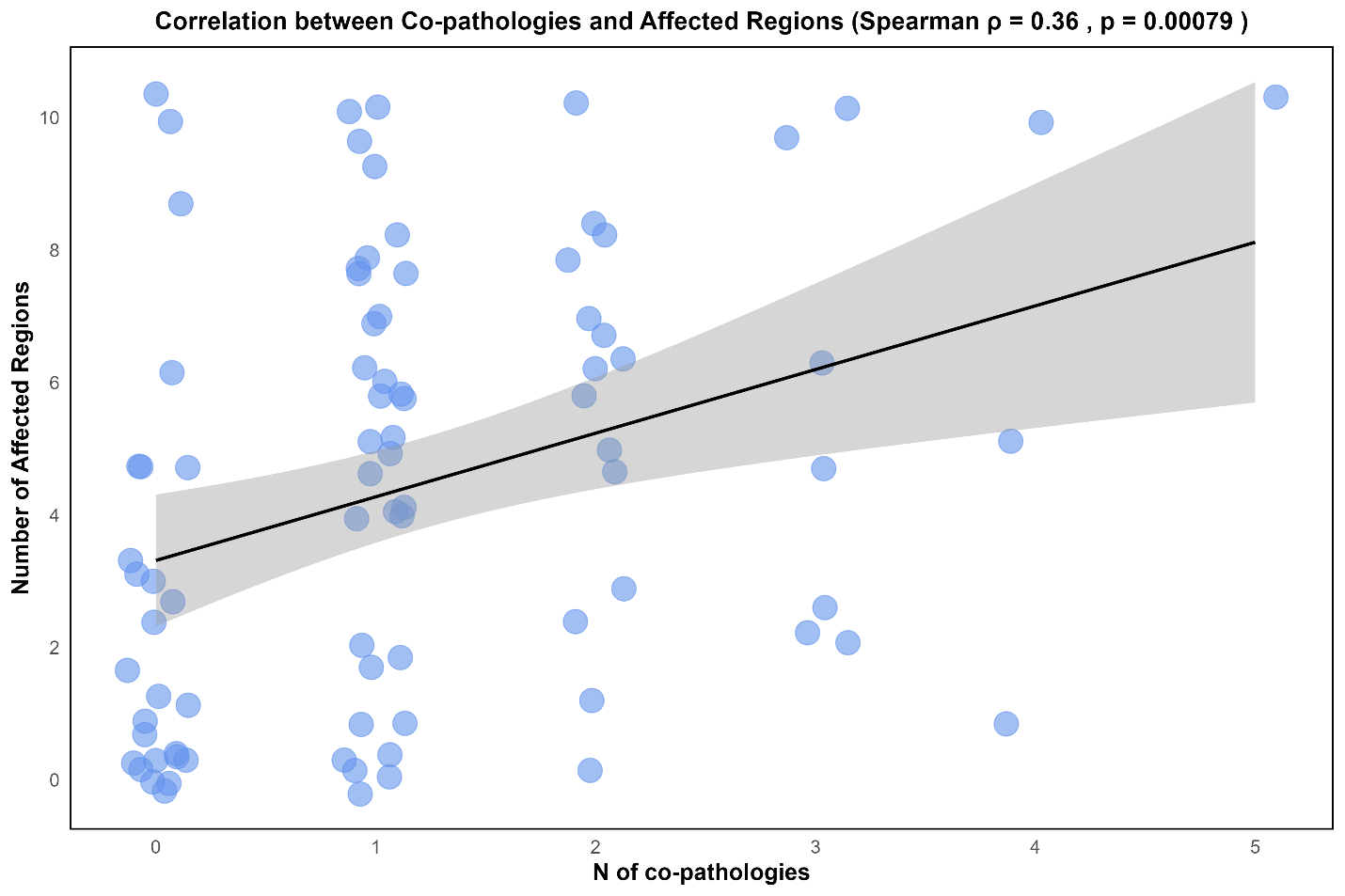


**Supplemental Figure 3.** Linear regression analysis outputs. **A.** Significant drivers of medial temporal SUVR changes included AGD (p<0.001 in the left temporal lobe; p=0.018 in the right temporal lobe) and TDP-43 (p=0.012 in the left temporal lobe). **B.** A synergistic interaction between AGD and TDP-43 was significant (p=0.003 in the left temporal lobe; p=0.032 in the right temporal lobe), highlighting their combined impact on metabolic alterations.


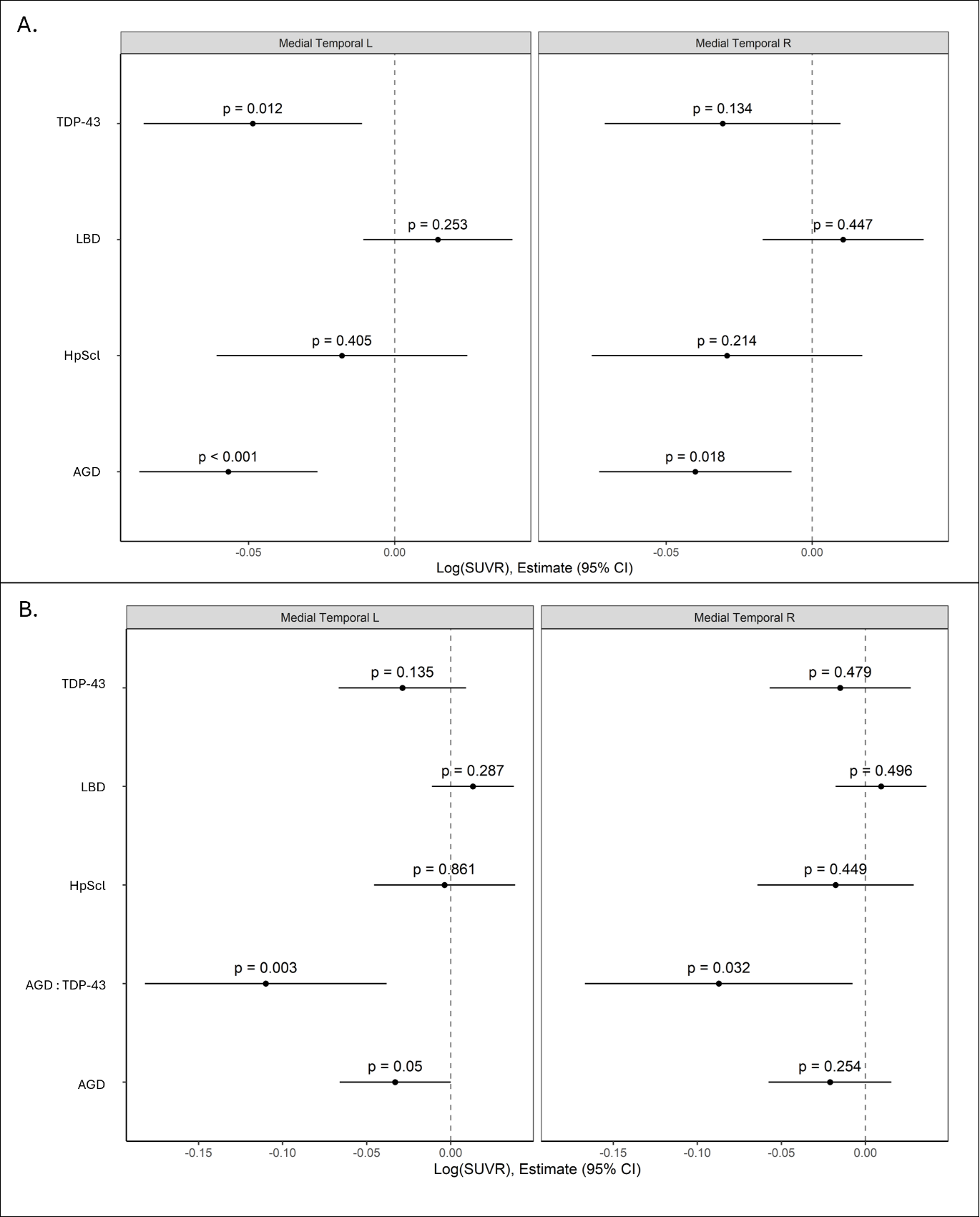

Supplement: Supplementary file 1 — Supporting Information [file ALZ-21-e70568-s001.docx]
